# Educating the Future of Science and Medicine

**DOI:** 10.3390/vetsci5020034

**Published:** 2018-03-23

**Authors:** Mark A. Brown

**Affiliations:** 1 Department of Clinical Sciences, Colorado State University, Fort Collins, CO 80523-1052, USA; Mark.Brown@colostate.edu; Tel.: +1-970-491-5782; 2 Epidemiology Section, Colorado School of Public Health, Fort Collins, CO 80523-1052, USA; 3 Institute for Learning and Teaching, Colorado State University, Fort Collins, CO 80523-1052, USA

**Keywords:** veterinary education, best practices, assessment

## Abstract

For the past two decades, veterinary educators have been at the forefront of innovations in educational practices related to science and medicine. Many of the resulting methods have been translated and implemented as best practices across the breadth of disciplines in higher education. However, past World Organization for Animal Health (OIE) global conferences have highlighted the necessity for improving global harmonization of veterinary medical education. This underscores a growing need for even broader dissemination of best practices and assessment programs related to educating our veterinary workforce.

## 1. Introduction

Innovations in veterinary medical education have revolutionized higher education practices across a breadth of academic disciplines. Veterinary programs have championed such high-impact practices as the *flipped classroom* [1] and *problem-based learning* [2], and they have paved the way for faculty in higher education to be appropriately assessed and rewarded for their teaching effort [3]. Beyond the immediate benefits of such innovations in professional veterinary medical programs, these have been particularly impactful in undergraduate science, technology, engineering, and mathematics (STEM) education [4,5,6], where historical adherence to traditional classroom methods and assessment of faculty based primarily on research productivity had stalled efforts to improve measures of persistence, retention, diversity, and academic performance. Despite these contributions, a recent call for global harmonization of veterinary medical education highlights the growing need for broader dissemination of such innovations for the benefit of veterinary education and, ultimately, the education our broader workforce across the sciences. 

## 2. Discussion 

**Figure 1:**
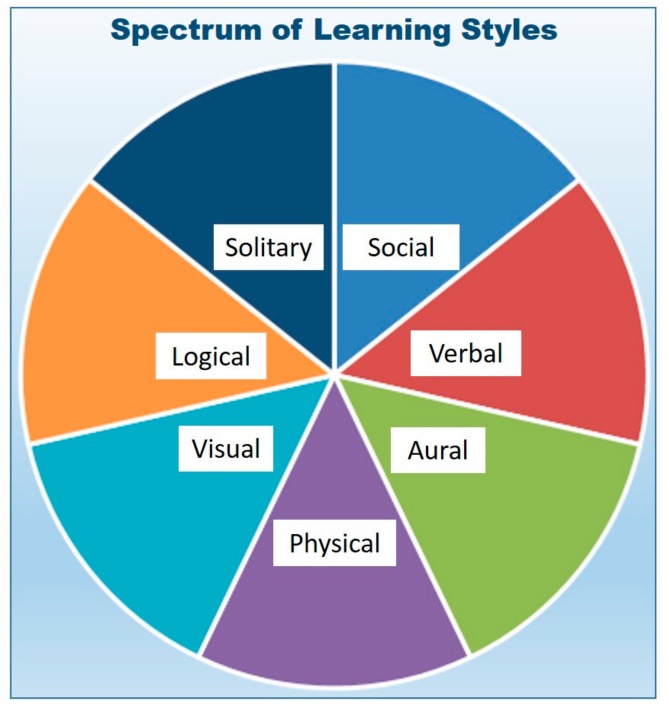
Spectrum of Learning Styles.

Research in the fields of medical and STEM education emphasizes the need to address the increasingly diverse learning requirements for students of science and medicine [7,8,9,10,11,12]. Every individual receives, interprets, and stores information according to a unique combination of experiential and innate learning styles. As a result, there is a variable reliance upon a range of factors in a broad learning spectrum [13,14,15,16] as demonstrated in Figure 1. Consequently, *like a thumbprint*, every learner processes incoming information based on their own distinct reliance on factors across their unique learning spectrum such that no two individuals learn in the exact same way. However, the traditional classroom relied upon a traditional lecture structure that reached only a subset of learning styles. Thus, historically, some students were required to seek external help from tutors and other learning resources if they wanted to better match their unique learning styles. These outside resources were often poorly matched to the classroom content and, as a result, a subset of students often failed to successfully navigate the complexities of challenging medical and STEM curricula. This led to diminished academic performance and disproportionately high rates of attrition in science and medical programs. 

The Flipped Classroom style of teaching has been embraced by most veterinary medical programs for its capacity to engage students with diverse learning needs [1,17,18,19]. This classroom model *engages* participants in a mixed style of learning that “flips” the traditional lecture paradigm by providing the instructional matter outside of the classroom often by an online medium. Applications of the instructional content, like problem sets, are then performed during the scheduled class time. This commonly occurs in the form of group assignments, such that each group member is afforded the opportunity to contribute and participate according to their own learning style. The instructor is then able to work with each student based on their distinct learning requirements. In many veterinary programs, flipped classrooms allow students to observe online lectures at their leisure and later illustrate their ability to apply course content in cooperation with other students under the guidance of their professor. This model has a proven ability to achieve higher academic performance, student understanding, applicability, and retention [18,20,21]. Thus, it is no surprise that this innovation has been so well embraced and implemented in veterinary education in a way that its impact and value can be observed and shared for the broader benefit of science and medical education. Just ten years ago, few faculty had even heard of the flipped classroom. Yet, in a recent national survey, some 55% of higher education faculty indicated that they had either flipped or were in the process of flipping their classrooms [22].

Another teaching method that has been successfully validated in veterinary education and applied more broadly is the critical review process. This is an essential element of higher education in the sciences and, in the professional world, it often contributes to a peer review system that serves as one of the *pillars of research integrity*. Research related to students who drop out of academic programs often reports that those students did not feel *engaged* in their academic environments [7,23,24]. Experiential learning and the critical review process have been used to enhance educational outcomes by providing students with practical problem-solving skills through problem-based learning [2,25]. The students’ direct exposure, review, and response to real-world applications provide a means for the development of complex, career-oriented skills. This method of learning is exceptionally suited for connecting difficult subjects across multiple disciplines [11,12,13,14,15,26]. However, veterinary educators have emphasized the importance of quality learning in this regard rather than overloading students with content. It is better to allow students to consider one application in a robust problem-based experience rather than have them execute multiple applications with little regard for rigor [27]. The most successful implementation of this method embeds engagement within a multi-layer methodology including faculty mentoring, interdisciplinary experiential learning, and skillset development.

Communication is a subject that has been overlooked in STEM disciplines for decades. However, its essential nature in medical practice has afforded it a central theme throughout veterinary education and this is finally having a ripple-effect throughout STEM education. Historically, STEM training has been communicated as matters of fact and theory and it has uniformly avoided emotional issues or matters of passion. The medical profession, however, does not have this luxury. Instead, clinicians find themselves face-to-face with human nature, demanding clients, and life-and-death situations. Thus, veterinary programs have routinely encouraged their students to consider the human and emotional elements of practicing medicine [28]. This need for developing clinicians who can respond to the passions and unpredictable factors of human situations has led to team-based approaches in veterinary education where students are forced to deal with the individual limitations of their fellow classmates [29]. In a team where everyone has an equal voice, a veterinary student cannot hide behind their knowledge of scientific theory and clinical skills. Rather they must strategize for the inclusion of human strengths and limitations and navigate the often-precarious issues of human interactions. In the past decade, lessons learned in this approach to clinical education have been applied to the broader STEM disciplines where they are steadily gaining traction resulting in better communicators among the STEM professions. 

Teaching effort has long been undervalued in STEM disciplines where acquisition of external funding and publication of peer reviewed research largely defines the outcome of tenure and promotion decisions. However, in professional medical education, there is acute awareness of the quality of teaching by virtue of student board results and professional success. That these outcomes ultimately impact program rankings has been the impetus for increasing consideration of quality of faculty teaching and a corresponding emphasis on rewarding teaching effort in the tenure and promotion process [3]. Greater care is now being given to calibrating teaching effort and to evaluating outcomes based not only on exam scores and student satisfaction, but also via peer teaching evaluations and longitudinal outcomes. Once again, this evolving emphasis on teaching has spilled-over into related biomedical disciplines and, from there, is gaining momentum across a range of basic STEM fields. 

## 3. Conclusions

Highlighted above are just a few of the educational practices for which veterinary medical programs have been at the forefront of innovation. To remain a driver in high impact education across both clinical and broader STEM disciplines, it is our responsibility to support the broader dissemination of best practices and assessment programs related to educating our veterinary workforce. To support this endeavor, *Veterinary Sciences* is currently accepting papers for a special issue, “Educating the Future of Veterinary Science and Medicine” (http://www.mdpi.com/journal/vetsci/special_issues/EFVSE). Therein, the journal invites papers related to: teaching innovations; best teaching practices; high impact practices; methods for better assessing students, programs, and faculty; educational research; case studies; and models for educating the future of veterinary medicine.
